# Ferredoxin 1: a gatekeeper in halting lung adenocarcinoma progression through activation of the GPRIN2 signaling pathway

**DOI:** 10.1186/s12967-024-05277-6

**Published:** 2024-05-27

**Authors:** Ming Liu, Shaoxian Wu, Haoyu Wu, You Zhou, Xinyu Zhang, Dawei Zhu, Jingting Jiang

**Affiliations:** 1 Department of Tumor Biological Diagnosis and Treatment Center, The Third Affiliated Hospital of Soochow University, Changzhou, 213003 Jiangsu China; 2 Department of Respiratory and Critical Care Medicine, The Affiliated Changzhou No.2 People’s Hospital of Nanjing Medical University, Changzhou, China; 3 Changzhou Medical Center, Nanjing Medical University, Changzhou, China; 4 Dalian Medical University, Dalian, China

**Keywords:** Lung adenocarcinoma, Cuproptosis, FDX1, GPRIN2

## Abstract

**Background:**

Lung adenocarcinoma (LUAD) is a highly lethal form of lung cancer. Despite advancements in treatments, managing LUAD is still challenging due to its aggressive behavior. Recent studies indicate that various molecular pathways, including the dysregulation of ferredoxin 1 (FDX1), play roles in LUAD progression. FDX1, a crucial protein in cellular redox reactions and energy metabolism, has been linked to several cancers. However, its exact role in the development of LUAD is not yet fully understood.

**Methods:**

We investigated the role of ferredoxin 1 (FDX1) in LUAD progression through analysis of its expression in LUAD tissues and its impact on patient survival. Functional assays were performed to assess the effects of FDX1 overexpression on LUAD cell proliferation, migration, and invasion. A xenograft model was employed to evaluate the tumorigenesis potential of LUAD cells with FDX1 overexpression. Mechanistic insights into FDX1 regulation were gained through depletion experiments targeting the G protein-regulated inducer of neurite outgrowth 2 (GPRIN2)/PI3K signaling pathway.

**Results:**

FDX1 expression was down-regulated in LUAD tissues, correlating with shorter patient survival. Overexpression of FDX1 suppressed LUAD cell proliferation, migration, and invasion in vitro, and inhibited tumorigenesis in vivo. Mechanistically, the GPRIN2/PI3K signaling pathway was implicated in FDX1 regulation, as depletion of GPRIN2 reversed the effects of FDX1 overexpression on cellular functions.

**Conclusions:**

Our findings highlight FDX1 as a potential tumor suppressor in LUAD, acting through modulation of the GPRIN2/PI3K signaling pathway. These results suggest FDX1 as a promising therapeutic target for LUAD treatment, warranting further investigation into its clinical relevance.

**Supplementary Information:**

The online version contains supplementary material available at 10.1186/s12967-024-05277-6.

## Introduction

Non-small cell lung cancer (NSCLC) constitutes approximately 85% of all lung cancer cases [1]. Emerging from the cells lining the lung airways, it transforms into a malignant tumor that, if untreated, can metastasize to other parts of the body. The often asymptomatic nature of NSCLC in its early stages poses challenges for timely detection and diagnosis. Symptoms such as coughing, shortness of breath, chest pain, and weight loss may manifest in later stages [2]. Risk factors encompass smoking, exposure to environmental pollutants, family history, and genetic mutations. Treatment modalities for NSCLC vary based on the cancer stage and may involve surgery, radiation therapy, chemotherapy, targeted therapy, or immunotherapy. Despite therapeutic strides, NSCLC remains a leading cause of global cancer-related mortality.

Lung adenocarcinoma (LUAD), a subtype of NSCLC and the most prevalent form of lung cancer, represents about 40% of all lung cancer cases [1]. Predominantly originating in the lung’s outer regions, LUAD manifests as the development of malignant tumors in glandular cells responsible for mucus and other secretions. Unlike other NSCLC subtypes, LUAD tends to exhibit a slower growth pattern and a higher likelihood of distant metastasis. Symptoms include coughing, shortness of breath, chest pain, and weight loss. Its risk factors mirror those of NSCLC, involving smoking, environmental pollutants, family history, and genetic mutations. Treatment strategies for LUAD, contingent on the cancer stage, encompass surgery, chemotherapy, targeted therapy, or immunotherapy. Despite therapeutic advancements, LUAD continues to be a leading cause of cancer-related mortality worldwide.

Ferredoxin 1 (FDX1) is a pivotal member within the ferredoxin protein family, essential for orchestrating iron-sulfur cluster (ISC) biogenesis, heme synthesis, and electron transfer reactions [3]. Recent studies have shed light on FDX1’s critical involvement in cuproptosis, a regulated form of cell death triggered by excessive copper levels [4]. Cuproptosis unfolds through the disruption of copper homeostasis, instigating mitochondrial dysfunction and oxidative stress [5]. Cellular accumulation of dysregulated copper sparks the generation of reactive oxygen species (ROS), culminating in structural damage and eventual cell demise [6].

FDX1 emerges as a key player in regulating both copper homeostasis and cuproptosis [4, 7]. Its interaction with the copper chaperone for superoxide dismutase, a protein facilitating copper transport into mitochondria for cellular respiration and antioxidant defense, underscores its multifaceted role [8]. FDX1 modulates the activity of copper chaperone for SOD-1 (CCS), influencing copper availability for mitochondrial functions [9]. In scenarios of copper overload, FDX1 facilitates the sequestration of copper away from mitochondria, averting its accumulation and diminishing ROS production [10]. The intricate involvement of FDX1 in cuproptosis underscores the complexity of regulatory networks governing copper homeostasis and their impact on cellular well-being and pathology.

Deeper exploration into the molecular mechanisms underpinning FDX1-mediated control of copper metabolism and cuproptosis can unveil promising therapeutic avenues for conditions associated with dysregulated copper homeostasis. In the present study, we meticulously examined the expression profile of FDX1 in both LUAD tissues and cell lines, uncovering a consistent down-regulation in these contexts. Notably, the overexpression of FDX1 emerged as a potent activator of cuproptosis, concurrently exerting inhibitory effects on the proliferation, migration, and invasion of LUAD cells, both in vitro and in vivo. Transcriptome sequencing brought to light that FDX1 played a pivotal role in elevating the expression of G protein-regulated inducer of neurite outgrowth 2 (GPRIN2) while concurrently suppressing phosphatidylinositol 3-kinase (PI3K) signaling. Strikingly, the depletion of GPRIN2 robustly reversed the effects induced by FDX1 overexpression, particularly in terms of inhibiting proliferation, migration, and invasion in LUAD cells.

## Materials and methods

### Cell lines and cell culture

The human LUAD cell lines A549 and H1975 were procured from the Chinese Academy of Sciences, Shanghai Institutes for Biological Sciences. Cells were maintained in DMEM supplemented with 10% fetal bovine serum (FBS) under standard culture conditions (5%CO_2_, 37°C).

### FDX1 expression levels in GEO database and TCGA database

LUAD microarray data (GES33532) was downloaded from the GEO database (http://www.ncbi.nih.gov/geo). The raw data were downloaded as MINiML files. Box plots are drawn by boxplot, the R software ggord package was used to draw PCA plot. RNA-sequencing expression (level 3) profiles and corresponding clinical information for FDX1 in lung adenocarcinoma tissues were downloaded from the TCGA dataset (https://portal.gdc.com). Relationship between FDX1 and GPRIN2 expression level and clinicopathological variables and in lung adenocarcinoma patients were shone in Tables 1 and 2. The measurement data are displayed as the mean ± SD. Unpaired t-test was used for analyzing statistical assessments. The association between FDX1 and clinical characteristic variables was analyzed using Pearson chi-squared test or Fisher’s exact test. The two-gene correlation map is realized by the R software package ggstatsplot, and the multi-gene correlation heatmap is displayed by the R software package.

**Table 1:** Relationship between FDX1 expression level and clinic-pathological variables and in lung adenocarcinoma patients

	Clinico-pathological variables	High FDX1	Low FDX1	*P*_value
Status	Alive	149	180	
	Dead	109	78	0.006
Age	Mean (SD)	66 (9.8)	64.7 (10.2)	
	Median [Min–Max]	67 [40–87]	65.5 [33–88]	0.145
Gender	Female	138	140	
	Male	120	118	0.93
Race	American Indian	1		
	Asian	3	5	
	Black	23	29	
	White	187	202	0.743
pT_stage	T1	29	38	
	T1a	19	28	
	T1b	23	32	
	T2	102	67	
	T2a	37	45	
	T2b	11	16	
	T3	23	24	
	T4	14	5	
	TX		3	0.014
pN_stage	N0	153	179	
	N1	52	44	
	N2	48	26	
	NX	5	6	
	N3		2	0.025
pM_stage	M0	186	161	
	M1	11	7	
	M1a	1	1	
	M1b	1	4	
	MX	57	83	0.054
pTNM_stage	I	1	4	
	IA	53	78	
	IB	73	67	
	IIA	22	28	
	IIB	39	32	
	IIIA	46	27	
	IIIB	6	5	
	IV	13	13	
	II		1	0.066
New_tumor_event_type	Metastasis	31	30	
	Metastasis: primary	1		
	Metastasis: recurrence	5	3	
	Primary	6	5	
	Recurrence	16	32	0.182
Smoking	Non-smoking	44	31	
	Smoking	204	223	0.106
Radiation_therapy	Non-radiation	52	90	
	Radiation	6	7	0.704
History_of_neoadjuvant_treatment	Neoadjuvant	3		
	No neoadjuvant	254	258	
Therapy_type	Ancillary: chemotherapy	2	1	
	Chemotherapy	76	79	
	Chemotherapy:	2	1	
	Chemotherapy: immunotherapy	3	1	
	Chemotherapy: other. specify in notes	1		
	Chemotherapy: other. specify in notes: targeted molecular therapy	2		
	Chemotherapy: targeted molecular therapy	4	2	
	Immunotherapy	1		
	Targeted molecular therapy	1	1	
	Vaccine		1	0.8

**Table 2:** Relationship between GPRIN2 expression level and clinicopathological variables and in lung adenocarcinoma patients

	Clinico-pathological variables	High GPRIN2	Low GPRIN2	*P*_value
Status	Alive	181	148	
	Dead	77	110	0.003
Age	Mean (SD)	65.9 (10)	64.7 (10.1)	
	Median [Min–Max]	67 [39–86]	65 [33–88]	0.16
Gender	Female	146	132	
	Male	112	126	0.251
Race	American Indian		1	
	Asian	5	3	
	Black	23	29	
	White	192	197	0.584
pT_Stage	T1	40	27	
	T1a	24	23	
	T1b	31	24	
	T2	77	92	
	T2a	37	45	
	T2b	17	10	
	T3	23	24	
	T4	7	12	
	TX	2	1	0.34
pN_Stage	N0	180	152	
	N1	43	53	
	N2	27	47	
	NX	1	1	
	N3	6	5	0.064
pM_Stage	M0	166	181	
	M1	9	9	
	M1a	2	3	
	M1b	75	65	
	MX	2		0.684
pTNM_Stage	I	3	2	
	IA	75	56	
	IB	72	68	
	IIA	22	28	
	IIB	36	35	
	IIIA	30	43	
	IIIB	3	8	
	IV	13	13	
	II	1		0.299
new_tumor_event_type	Metastasis	30	31	
	Metastasis: primary	3	5	
	Metastasis: recurrence	8	3	
	Primary	21	27	
	Recurrence	1		0.332
Smoking	Non-smoking	42	33	
	Smoking	210	217	0.335
Radiation_therapy	Non-radiation	76	66	
	Radiation	5	8	0.453
History_of_neoadjuvant_treatment	Neoadjuvant		3	
	No neoadjuvant	258	254	
Therapy_type	Ancillary: chemotherapy	2	1	
	Chemotherapy	74	81	
	Chemotherapy:	1	2	
	Chemotherapy: immunotherapy	2	2	
	Chemotherapy: other. specify in notes		1	
	Chemotherapy: other. specify in notes: Targeted Molecular therapy	1	5	
	Chemotherapy: targeted molecular therapy	1	1	
	Immunotherapy	2		
	Targeted molecular therapy	1		
	Vaccine	1		0.706

### Immunohistochemistry (IHC) and evaluation of FDX1 immunostaining

To assess the expression of FDX1 in human LUAD tissues and adjacent normal tissues, an IHC assay was conducted following a previously established protocol [11–13]. The paraffin-embedded CC tissue MicroArray, acquired from Outdo (Shanghai Outdo Biotech Co., Ltd., Shanghai, China), underwent dewaxing in xylene, rehydration in graded ethanol solutions, and drying at 90 °C for 4 h. After blocking endogenous peroxidase activity with a 0.3% hydrogen peroxide solution for 15 min, sections were rinsed with PBS for 5 min and blocked with a 3% BSA solution at room temperature for 30 min. Subsequently, sections were incubated with the primary antibody (rabbit anti-human FDX1 monoclonal antibody, catalog No. 12592-1-AP, Poteintech, Wuhan, China, used at 1:200) at 4 °C overnight, followed by incubation with HRP-labeled secondary antibody at 37 °C for 30 min after PBS washing. Sections were then dehydrated, cleared, and mounted. Diaminobenzene served as the chromogen, and hematoxylin was used as the nuclear counterstain. The assessment of FDX1 immunostaining intensity employed the H-score method, calculated as follows: H-score = (%unstained tumor cells × 0) + (% weakly stained tumor cells × 1) + (%moderately stained tumor cells × 2) + (% strongly stained tumor cells × 3). H-scores ranged from 0 (100% negative tumor cells) to 300 (100% strongly stained tumor cells).

### FDX1 over-expression and GPRIN2 knockdown lentiviral vectors construction and infection

The FDX1 overexpression plasmid, constructed using the pLVX-puro lentiviral vector and targeting FDX1 (NM_004109.5, GenBank), was generated. GPRIN2 expression was knocked down in human lung cancer cell lines A549 and H1975 using RNAi methodology. Shanghai Genelily Biotech Co., Ltd. provided the pLVX-puro-FDX1 lentiviral vector and Small hairpin RNA (shRNA) against the human GPRIN2 gene (NM_001385282.1, GenBank). The shRNA sequence targeting FDX1 was: 5′-GAGGATGAGACTTCTAACTTTCAAGAGAAGTTAGAAGTCTCATCCTCTTTTTT-3′. Co-infection of Lipofectamine 2000 (Invitrogen Life Technologies) with either pLVX-puro-FDX1 or pLVX-shRNA2-shFDX1, alongside psPAX2 (Addgene, Inc.) and pMD2.G (Addgene, Inc.), was performed in 293T cells. After a six-hour incubation, the 293T cells were cultured in complete media (Gibco, 10% FBS) instead of DMEM. Supernatant was collected and concentrated using a 0.22 μm PES filter after an additional 48 h of incubation. A549 and H1975 cells were infected with the packed lentiviral supernatant of pLVX-puro-FDX1 and pLVX-shRNA2-shFDX1. Following infection, the medium was replaced with fresh media after 6 h, and subsequent stability screening was conducted 48 h later on A549 and H1975 cells.

### RNA extraction and quantitative real-time PCR (qRT-PCR)

RNA extraction and qRT-PCR were conducted following established protocols [13]. Briefly, qRT-PCR was performed on an ABI VII7 system (Applied Biosystems, USA) utilizing SYBR Green as a DNA-specific fluorescent dye. GAPDH was selected as the housekeeping gene. The primer sequences of the housekeeping gene (GAPDH) and the target genes (FDX1 and GPRIN2) were as follows: GAPDH forward primer: 5′-TGACTTCAACAGCGACACCCA-3′, GAPDH reverse primer: 5′- CACCCTGTTGCTGTAGCCAAA-3′, FDX1 forward primer: 5′-TGCATGTGAGGGAACCCTGG -3′, FDX1 reverse primer: 5′-ATTTGGCAGCCCAACCGTGA -3′, GPRIN2 forward primer: 5′- AGCAGCACTGTGGGCAATGT -3′, GPRIN2 reverse primer: 5′-CCTCAGGAGCCAGGTCCCTT -3’. The relative expression of the target genes was calculated using the comparative Ct method (2^−ΔΔCt^).

### Western blotting analysis

Western blotting analysis was carried out using previously outlined procedures, employing anti-FDX1 (1:1000, Catalog No. 12592-1-AP, Proteintech, Wuhan, China) and HRP-labeled anti-GAPDH (1:5000, Kancheng Biotechnology, Shanghai, China). The immunoreactive bands were visualized using an enhanced chemiluminescence detection kit (Thermo Fisher, MA, USA). Densitometry, conducted using a video documentation system (Gel Doc 2000, Bio-Rad), enabled quantification of band densities.

### Cell proliferation, migration, and invasion assays

Cell proliferation was assessed using the Cell Counting Kit-8 (CCK-8), while cell migration was evaluated through a wound healing assay. The invasive ability of cells was examined using Matrigel (BD Bioscience, San Diego, CA)-coated Transwell chambers (8-μm pore size, BD Bioscience, San Diego, CA), as previously outlined.

### RNA-seq

RNA-Seq was conducted to identify potential FDX1-regulated mRNA, following a previously described protocol [13]. Transcriptome reads from RNA-Seq experiments were aligned to the reference genome (hg19) using Hisat2 software. Gene expression levels were quantified using the Ballgown package. A statistical significance threshold of *P* < 0.05 was applied.

### Tumor xenografts

BALB/C nude mice (5 weeks old) obtained from the SLAC Animal Center in Shanghai, China, were utilized to establish xenograft tumor models. Approval for all animal experiments was obtained from the Animal Care and Use Committee of the Third Affiliated Hospital of Soochow University. LUAD models were established by subcutaneously injecting A549 cells (OE-NC and OE-FDX1) into nude mice. Tumor volume was assessed every 5 days using length and width measurements, calculated by the formula: Tumor volume = (length × width^2^)/2. After 30 days, mice were euthanized, and tumors were excised, photographed, and weighed.

### Statistical analysis

All experiments were conducted in triplicate, and statistical analyses were performed using GraphPad Prism 9.0. Data were presented as mean ± SEM in all figures. Student’s *t*-test, one-way ANOVA, and two-factor ANOVA were employed to compare differences between and among groups. Statistical significance was acknowledged at *P* < 0.05 (two-tailed).

## Results

### FDX1 Down-regulation in LUAD Tissues

**Fig. 1:**
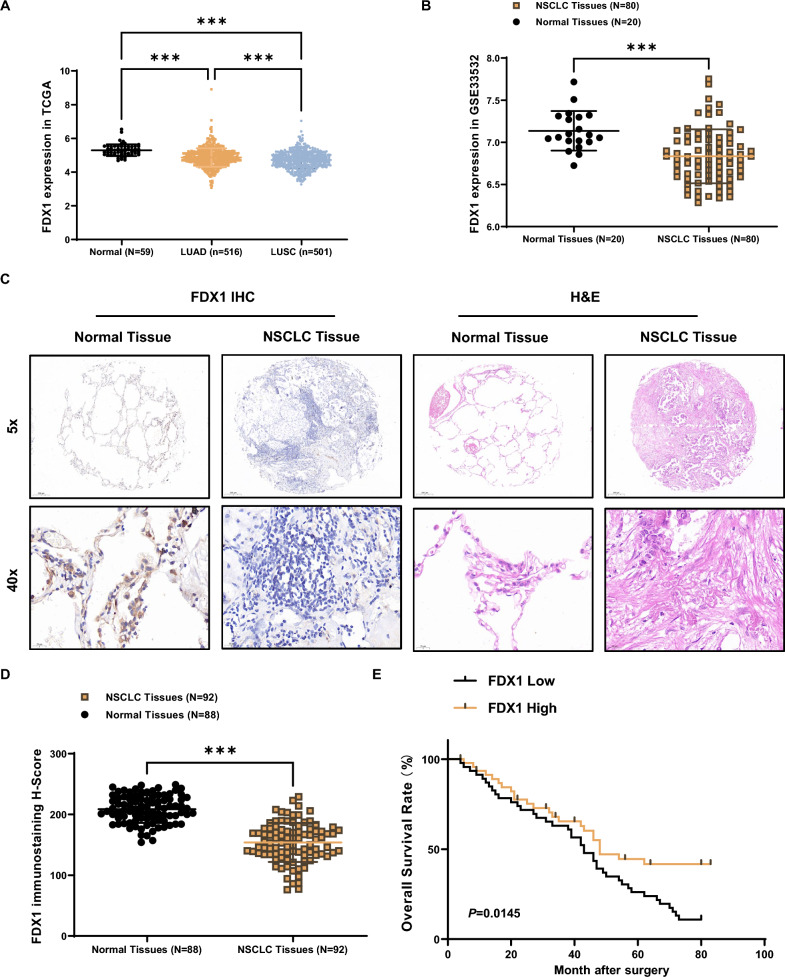
FDX1 is significantly downregulated in LUAD tissues. **A** Analysis of FDX1 expression in LUAD using the TCGA database. **B** Analysis of FDX1 expression in LUAD using the GEO database (GSE33532). **C** IHC analysis of FDX1 protein levels in LUAD tissue array. **D** H-score results. **E** Kaplan–Meier plot. ^***^P < 0.001

To elucidate the potential role of FDX1 in LUAD progression, we initially screened the TCGA database, revealing a significant down-regulation of FDX1 in LUAD tissues (n = 516) and LUSC tissues (n = 501) compared to normal tissues (n = 59) (Fig. 1A). Similarly, in GEO database (GSE33532), it showed a significant down-regulation of FDX1 in LUAD tissues (n = 80) compared to adjacent normal tissues (n = 20) (Fig. 1B). Subsequently, immunohistochemistry (IHC) in an LUAD tissue array comprising 92 human LUAD tissues and 88 paired adjacent normal tissues demonstrated that FDX1 protein levels were notably lower in the cytoplasm of LUAD tissues (Fig. 1C). The H-score from the IHC results (Fig. 1D) affirmed this observation, highlighting a significant reduction in FDX1 protein levels, and Kaplan–Meier plot analysis indicated that low FDX1 protein levels correlated with shorter patient survival (Fig. 1E). These findings collectively established that FDX1 was significantly down-regulated in LUAD tissues.

### FDX1 depletion and impaired functionalities in LUAD cell lines

**Fig. 2:**
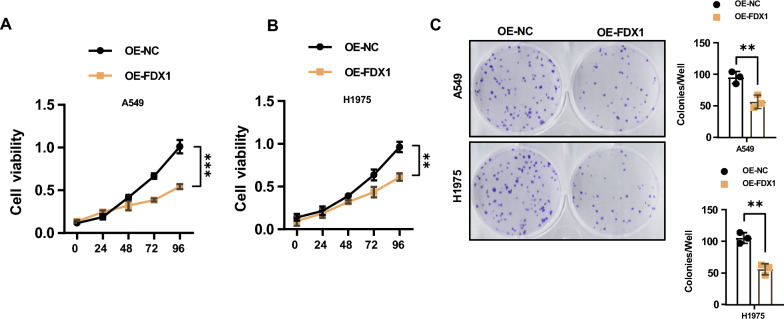
FDX1 depletion impairs the proliferation ability in LUAD cell lines. **A** and **B** CCK8 assay and **C** Colony formation assay were utilized to analyze the impact of FDX1 overexpression on the cell proliferation ability in A549 and H1975 cells; ^**^P < 0.01 and ^***^P < 0.001

**Fig. 3:**
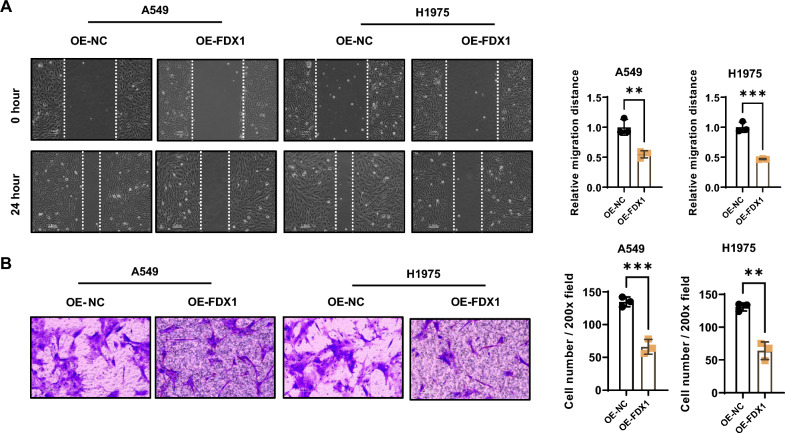
FDX1 depletion impairs the migration and invasion abilities in LUAD cell lines. **A** Wound healing assay evaluated the effect of FDX1 overexpression on the migration ability of A549 and H1975 cells. **B** Transwell invasion assay assessed the role of FDX1 overexpression on the invasion abilities of A549 and H1975 cells. ^**^P < 0.01 and ^***^P < 0.001

To comprehend the functional role of FDX1 in LUAD cells, we established FDX1 overexpression (OE) cell lines (A549 and H1975) through lentiviral transduction. qRT-PCR results demonstrated a significant elevation in FDX1 mRNA levels in the OE-FDX1 groups compared to the OE-NC groups in both A549 and H1975 cells (Supplementary Fig. 1A). Western blotting analysis (Supplementary Fig. 1B) and immunofluorescence staining (Supplementary Fig. 1C) further confirmed the increased FDX1 protein levels in the OE-FDX1 groups. CCK-8 assay revealed a substantial reduction in the cell proliferation ability in FDX1-overexpressing LUAD cells compared to vector control groups (Fig. 2A and B). Additionally, the colony formation assay illustrated significantly fewer colonies in FDX1-overexpressing LUAD cells (Fig. 2C). Furthermore, wound healing and invasion assays revealed a notable decrease in migration (Fig. 3A) and invasive capabilities (Fig. 3B) in A549 and H1975 cells with FDX1 overexpression. These results collectively suggested that FDX1 overexpression impaired the proliferation, migration, and invasion abilities of LUAD cells.

### Impaired tumorigenesis by FDX1 overexpression in the xenograft model

**Fig. 4:**
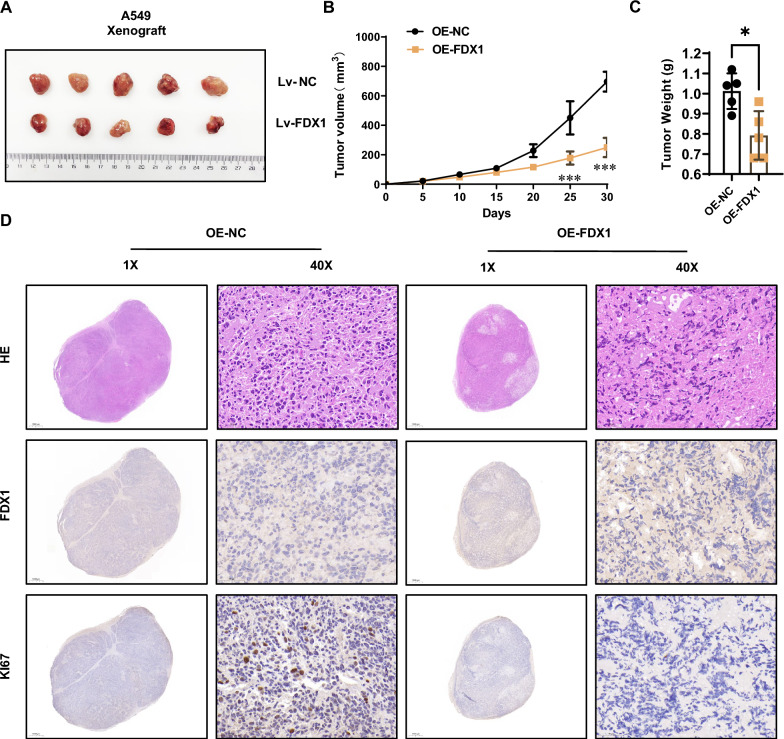
FDX1 overexpression impairs the tumorigenic ability of LUAD cells in the Xenograft model. **A** A subcutaneous transplantation tumor model assessed the impact of FDX1 overexpression on the tumorigenesis of A549 cells. The resulting tumors from A549 (Lv-NC and Lv-FDX1) cells were isolated and photographed. **B** A growth curve of tumors generated from A549 cells was plotted every 5 days after subcutaneous injection. **C** The weight of tumors generated from A549 cells was analyzed. **D** IHC staining depicted the Ki67-positive cells in the generated tumors. ^*^P < 0.05 and ^***^P < 0.001

To assess the impact of FDX1 on the tumorigenic potential of LUAD cells in vivo, we established a subcutaneous transplantation tumor model using FDX1- overexpressing A549 cells in nude mice. The resulting tumors, depicted in Fig. 4A, exhibited a smaller size in the FDX1 overexpression group. Notably, FDX1 overexpression significantly reduced tumor growth (Fig. 4B) and tumor weight (Fig. 4C). IHC staining revealed a substantial decrease in Ki67-positive cells in the generated tumors with FDX1 overexpression, further indicating a diminished tumorigenic ability of A549 cells (Fig. 4D). In summary, these results strongly suggested that FDX1 overexpression impaired the tumorigenic ability of LUAD cells in the Xenograft model.

### Elevated GPRIN2 signaling by FDX1 overexpression

**Fig. 5:**
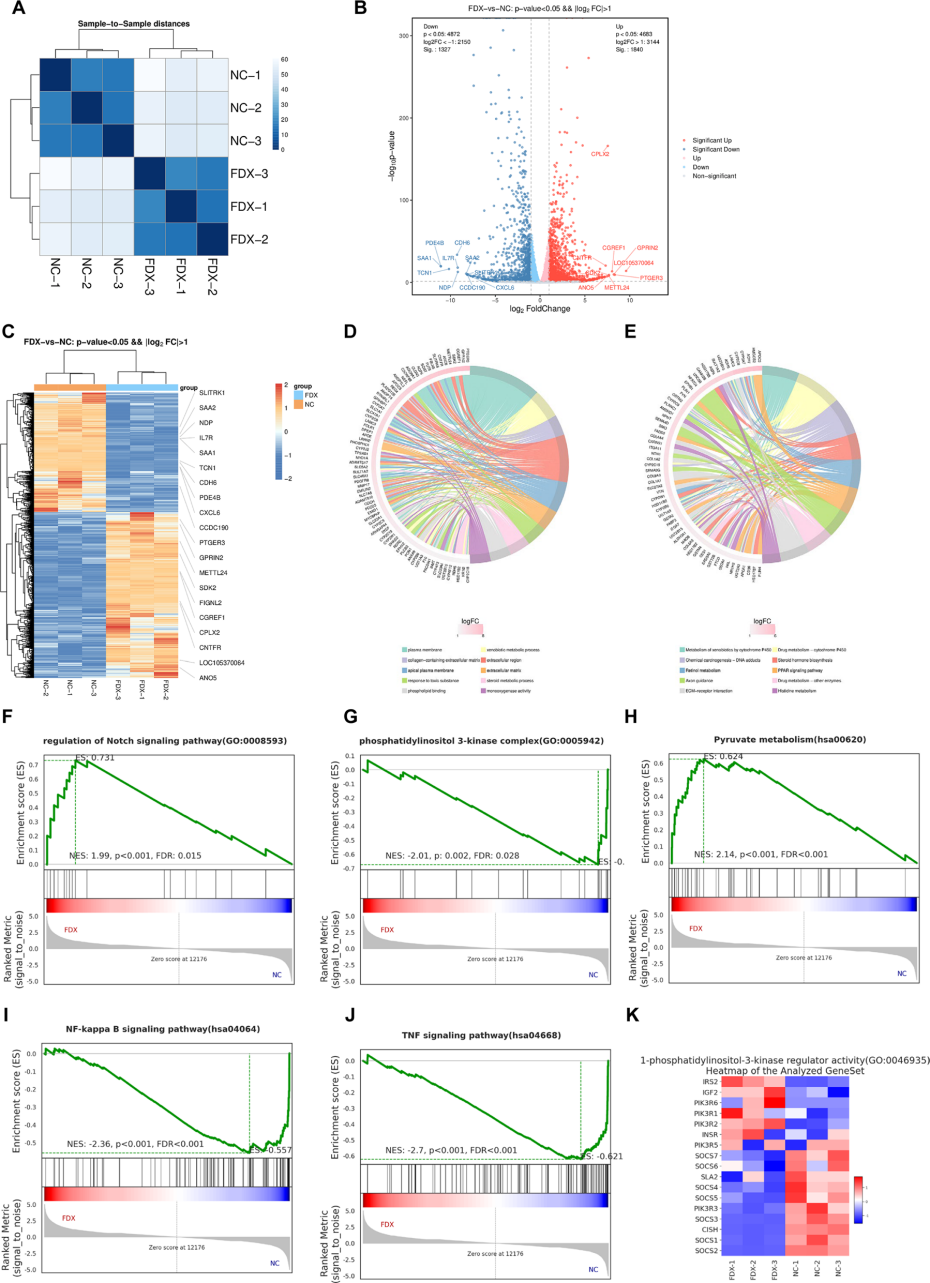
FDX1 overexpression elevates the GPRIN2 signaling. **A** Heat map displaying the hierarchically clustered Pearson’s correlation matrix resulting from comparing the expression level of each gene in the NC and FDX1-overexpression transcriptomes. **B** Volcano plot showing the expression change of genes between the NC and FDX1-overexpression transcriptomes, leading to the identified DEGs. Upregulated genes (FC ≥ 2; FDR < 0.05) are labeled in red, and downregulated genes (FC ≤  − 2; FDR < 0.05) are labeled in blue. **C** Hierarchical clustering of the expression levels of all the identified DEGs in NC and FDX1-overexpression samples. FPKM values are log2-transformed and then median-centered by each gene. **D** The top 10 GO biological processes of FDX1-upregulated and downregulated genes. **E** The top 10 KEGG pathways of FDX1-upregulated and downregulated genes. **F**–**J** GESA analysis was used to show several dysregulated signaling pathways. **K** Hierarchical clustering of the expression levels of PI3K signaling pathway regulators in the NC and FDX1-overexpressing A549 cells

To delve into the underlying regulatory mechanism of FDX1, we conducted a transcriptomic comparison between FDX1-overexpressing A549 cells and vector control cells. Six cDNA libraries were constructed and sequenced (three biological replicates for each group), and the raw reads from RNA-Seq were processed and mapped to the human genome. The resulting fragments per kilobase of transcript per million fragments mapped (FPKM) values were used to quantify gene expression levels. The correlation matrix of FPKM values indicated high consistency among all six transcriptome datasets, with a noticeable transcriptional difference induced by increased FDX1 expression (Fig. 5A).

The volcano map, based on edgeR analysis, delineated differentially expressed genes (DEGs), with 1,840 genes up-regulated and 1,327 genes down-regulated (Fig. 5B). A heatmap of DEGs illustrated a clear distinction between the FDX1 and NC groups, revealing the up-regulation of GPRIN2 in FDX1 overexpressing A549 cells (Fig. 5C). GEPIA database analysis confirmed lower GPRIN2 expression in LUAD tissues than in normal tissues, and patients with lower GPRIN2 expression exhibited shorter survival, paralleling the findings for FDX1 (Supplemental Fig. S2A and B). In GEO database (GSE33532), it showed a significant down-regulation of GPRIN2 in LUAD tissues (n = 80) compared to adjacent normal tissues (n = 20) (Supplemental Fig. 2C). Moreover, the expression of FDX1 and GPRIN2 showed a positive correlation in the LUAD tissues in GEO database (GSE33532) (Supplemental Fig. S2D) and TCGA database (Supplemental Fig. S2E).

Functional analysis using GO and KEGG demonstrated that FDX1-regulated genes were involved in various biological processes and pathways, with the top 10 up-regulated GO terms (Fig. 5D) and KEGG pathways (Fig. 5E) highlighted. GSEA analysis unveiled the inhibition of several signaling pathways by FDX1, including Notch, PI3K, pyruvate metabolism, NF-κB, and TNF signaling pathways (Fig. 5F–J). Notably, regulators of the PI3K signaling pathway were predominantly affected in FDX1-overexpressing A549 cells (Fig. 5K), suggesting that GPRIN2/PI3K signaling might be the critical pathway regulated by FDX1.

### GRPIN2-Dependent Impairment of Cellular Functions by FDX1 Overexpression in LUAD Cell Lines

**Fig. 6:**
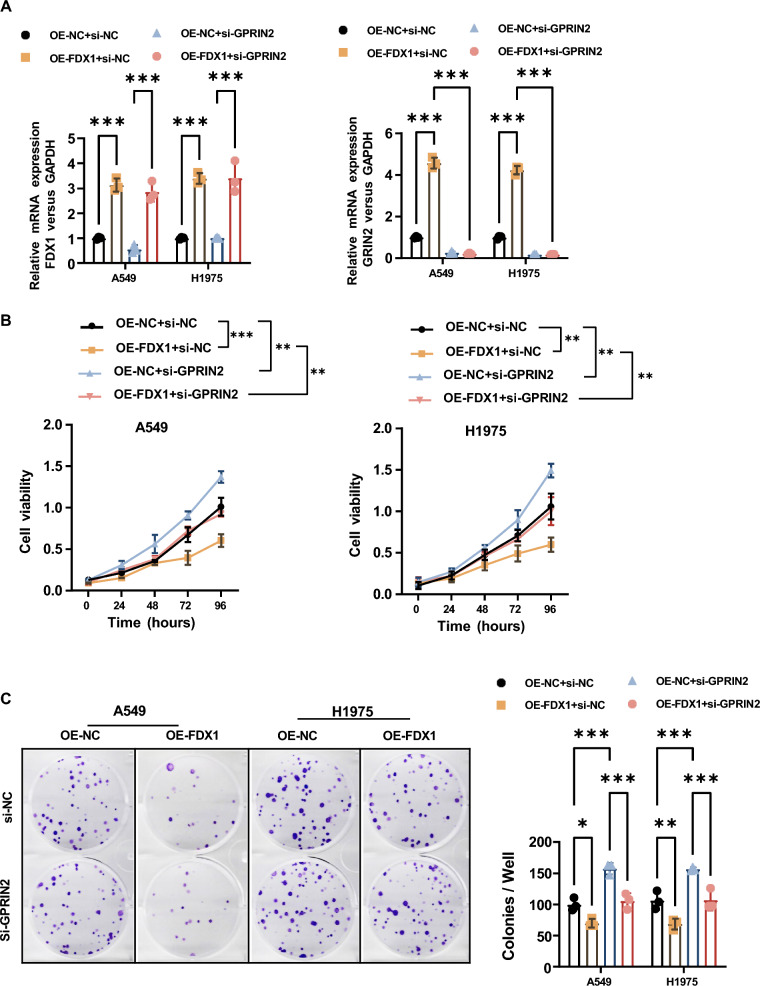
FDX1 overexpression impairs the proliferation ability in LUAD cell lines in a GRPIN2-dependent manner. **A** qRT-PCR was used to verify the overexpression efficiency of FDX1 and the depletion efficiency of GPRIN2 in A549 and H1975 cells. **B** CCK8 assay and **C** colony formation assay were used to analyze the effect of FDX1 overexpression with or without GPRIN2 depletion on the cell proliferation ability in A549 and H1975 cells. ^*^P < 0.05, ^**^P < 0.01 and ^***^P < 0.001

**Fig. 7:**
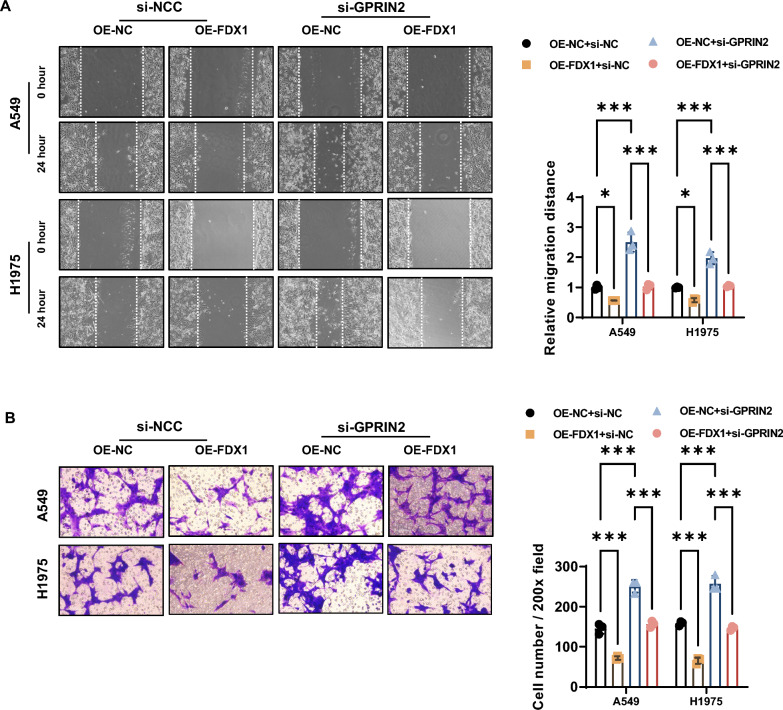
FDX1 overexpression impairs the migration and invasion abilities in LUAD cell lines in a GRPIN2-dependent manner. **A** The effect of FDX1 overexpression with or without GPRIN2 depletion on the migration ability of A549 and H1975 cells was evaluated using a wound healing assay. **B** The role of FDX1 overexpression with or without GPRIN2 depletion on invasion abilities of A549 and H1975 cells was analyzed by a Transwell invasion assay. ^*^P < 0.05 and ^***^P < 0.001

To elucidate the pivotal role of GPRIN2 in the FDX1 overexpression-mediated alterations in cellular functions of LUAD cells, we depleted GPRIN2 expression in both vector control and FDX1-overexpressing LUAD cell lines (A549 and H1975). The efficacy of GPRIN2 depletion was verified through qRT-PCR (Fig. 6A). Remarkably, GPRIN2 depletion resulted in enhanced cell proliferation and colony formation (Fig. 6B and C), migration (Fig. 7A), and invasion abilities (Fig. 7B) in LUAD cells. Furthermore, GPRIN2 depletion successfully reversed the inhibitory effects of FDX1 overexpression on cell proliferation (Fig. 6B and C), migration (Fig. 7A), and invasion abilities (Fig. 7B) in LUAD cells.

**Fig. 8:**
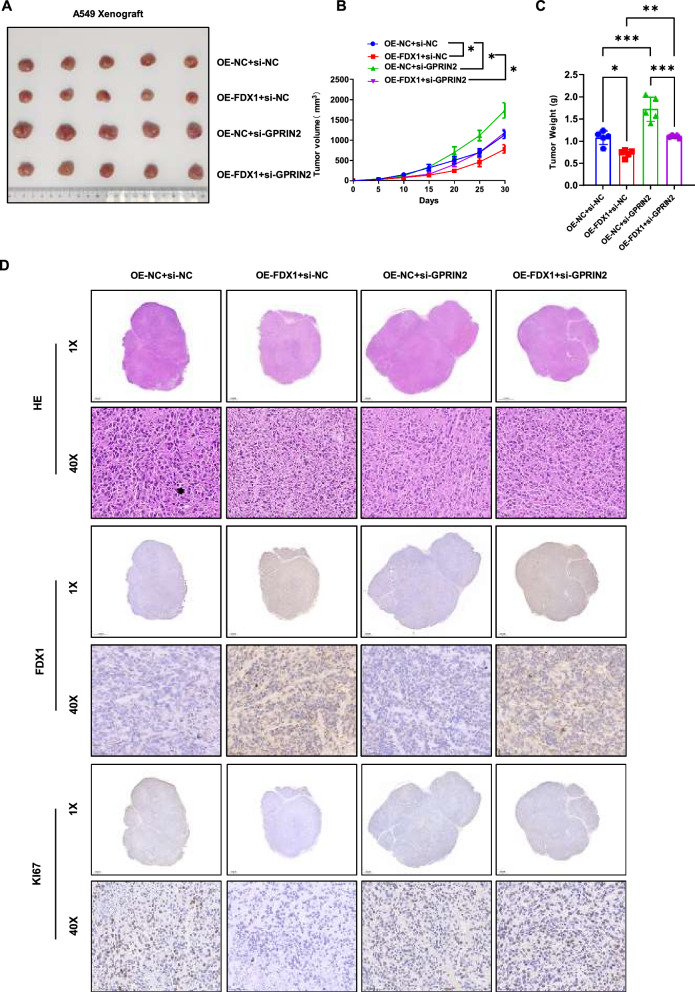
FDX1 overexpression impairs the tumorigenic potential of LUAD cells in a GRPIN2-dependent manner. **A** A subcutaneous transplantation tumor model was used to evaluate FDX1 overexpression on the tumorigenesis of A549 cells. The generated tumors from A549 (Lv-NC, Lv-FDX1, Lv-NC + siGPRIN2, and Lv-FDX1 + siGPRIN2) cells were isolated and pictured. **B** A growth curve of tumors generated from A549 cells was plotted every 5 days after subcutaneous injection. **C** The weight of tumors generated from A549 cells was analyzed. **D** IHC staining showed the Ki67-positive cells in the generated tumors. ^*^P < 0.05, ^**^P < 0.01 and ^***^P < 0.001

To evaluate the impact of GPRIN2 and FDX1 on the tumorigenic potential of LUAD cells in vivo, we established a subcutaneous transplantation tumor model using FDX1-overexpressing A549 cells with or without GPRIN2 knockdown in nude mice. The resulting tumors are depicted in Fig. 8A, revealing that GPRIN2 depletion significantly increased tumor growth (Fig. 8B) and tumor weight (Fig. 8C) in LUAD cells. Conversely, FDX1 overexpression led to a significant reduction in tumor growth (Fig. 8B) and tumor weight (Fig. 8C) in LUAD cells, an effect that was counteracted by GPRIN2 depletion. Consistently, the IHC assay demonstrated that FDX1 depletion markedly decreased the number of Ki67-positive cells in the generated tumors, indicative of reduced tumorigenic ability in A549 cells (Fig. 8D). GPRIN2 depletion substantially increased the number of Ki67-positive cells in both tumors generated using normal and FDX1-overexpressing A549 cells. Collectively, these results underscored that FDX1 overexpression impaired the proliferation, migration, and invasion abilities in LUAD cell lines in a GPRIN2-dependent manner.

## Discussion

The findings from this study offered valuable insights into the role of FDX1 in the progression of LUAD. A series of experiments were conducted to investigate the impact of FDX1 on various cellular functions and tumorigenic ability in LUAD cell lines. Several databases examining FDX1 expression in human cancers have identified FDX1 as a prognostic biomarker for pan-cancer. Higher FDX1 expression is associated with better disease-free survival (DFS) and overall survival (OS) in renal cancer, liver hepatocellular carcinoma, LUAD, and lung squamous cell carcinoma [14]. Furthermore, FDX1 expression is linked to specific immune subtypes in ACC, BRCA, KIRP, KIRC, LIHC, LGG, PRAD (prostate adenocarcinoma), SARC, STAD, THCA, and UCEC (uterine corpus endometrial carcinoma) [7, 15]. In this context, our study revealed a significant down-regulation of FDX1 in LUAD tissues compared to adjacent normal tissues. This observation was consistent with data obtained from the GEO database, as well as immunoblotting and IHC analyses.

Moreover, Kaplan–Meier plot analysis indicated that patients with low levels of FDX1 protein had significantly shorter survival, highlighting the potential prognostic value of FDX1 in LUAD. These comprehensive findings underscored the significance of FDX1 as a potential prognostic biomarker in the context of LUAD. Cuproptosis, also known as copper-induced cell death or ferroptosis-like cell death, is a recently identified form of regulated cell death linked to the dysregulation of copper metabolism [5, 16]. Copper, an essential trace element, plays a pivotal role in various biological processes such as cellular respiration, antioxidant defense, and enzymatic reactions [17]. However, an imbalance in copper homeostasis can result in cytotoxicity, triggering cuproptosis [15]. In this process, elevated copper levels disrupt cellular redox balance, leading to oxidative stress. Consequently, lipid peroxidation, mitochondrial dysfunction, and the accumulation of ROS occur in cells. The excessive production of ROS overwhelms cellular antioxidant defenses, causing damage to cellular structures and ultimately culminating in cell death [5, 18].

Cuproptosis has been implicated in various pathological conditions, including neurodegenerative diseases, liver diseases, and cancer [19]. Dysregulated copper metabolism and cuproptosis are evident in neurodegenerative disorders such as Alzheimer’s and Parkinson’s diseases, where the accumulation of copper in specific brain regions contributes to neuronal cell death [20, 21]. In the context of cancer, dysregulated copper transporters and copper chelation have shown promise in inducing cuproptosis as a potential therapeutic strategy [22–25].

FDX1 plays a crucial role in copper-dependent cell death by facilitating the transfer of electrons from NADPH to mitochondrial cytochrome P450 via ferredoxin reductase. Despite accumulating evidence supporting a vital role for FDX1 in tumorigenesis in certain cancers, its functional significance in cancers, particularly in LUAD, remains unclear. To comprehend the functional role of FDX1 in LUAD cells, FDX1 was overexpressed in A549 and H1975 cell lines. The study revealed that FDX1 overexpression impaired the proliferation, migration, and invasion abilities of LUAD cells, as evidenced by various assays, including CCK-8, colony formation, EdU, wound healing, and invasion assays. These results collectively suggested that FDX1 acted as a tumor suppressor in LUAD by inhibiting the aggressive behavior of cancer cells.

Moreover, the impact of FDX1 on tumorigenic ability was investigated in a xenograft model, demonstrating that FDX1 overexpression in A549 cells led to significant decreases in tumor growth and tumor weight in nude mice. IHC assay revealed a reduction in Ki67-positive cells, further supporting the inhibitory effect of FDX1 on tumorigenesis in LUAD. To uncover the underlying regulatory mechanism of FDX1, a transcriptomic comparison was conducted between FDX1-overexpressing A549 cells and control cells. The analysis revealed several dysregulated genes, with GPRIN2 emerging as upregulated in FDX1-overexpressing cells. Subsequent analysis using the GEPIA database indicated lower expression of GPRIN2 in LUAD tissues compared to normal tissues, and patients with lower GPRIN2 expression exhibited shorter survival. This finding suggested a potential association between FDX1 and GPRIN2 in LUAD progression.

To validate the role of GPRIN2 in FDX1-mediated effects, we depleted GPRIN2 expression in both control and FDX1-overexpressing cell lines. Intriguingly, GPRIN2 depletion enhanced cell proliferation, migration, and invasion abilities in LUAD cells, counteracting the inhibitory effects of FDX1 overexpression on these cellular functions. Additionally, in vivo experiments employing the xenograft model demonstrated that GPRIN2 depletion increased tumor growth and tumor weight, while FDX1 overexpression decreased both. This finding further substantiated the notion that FDX1 exerted its effects on LUAD through a GPRIN2-dependent mechanism.

The PI3K signaling pathway is a crucial cellular pathway involved in various biological processes, including cell growth, survival, metabolism, and migration [26]. Dysregulation of the PI3K pathway is implicated in numerous cancers, including NSCLC [27]. In NSCLC, the PI3K pathway is frequently aberrantly activated, contributing to tumor initiation, progression, metastasis, and therapy resistance.

Various mechanisms can lead to the activation of the PI3K pathway in NSCLC, including genetic alterations such as activating mutations in the PIK3CA gene, loss of the tumor suppressor PTEN (phosphatase and tensin homolog), amplification of the EGFR (epidermal growth factor receptor) gene, or activation of upstream receptor tyrosine kinases [28]. Activation of the PI3K pathway in NSCLC promotes cell survival and proliferation by stimulating downstream effectors such as Akt (also known as protein kinase B) [29]. Akt activation inhibits apoptosis and promotes cell cycle progression [30]. Moreover, the PI3K pathway regulates protein synthesis and metabolism through downstream targets, including mTOR (mammalian target of rapamycin), leading to increased cellular growth and proliferation. The PI3K pathway also plays a crucial role in mediating epithelial-mesenchymal transition (EMT), enhancing tumor cell invasion and metastasis [31]. Activation of the PI3K pathway induces the expression of EMT-inducing transcription factors and promotes cytoskeleton remodeling, enabling cancer cells to acquire a more mesenchymal-like phenotype [32].

Moreover, the PI3K pathway is implicated in the development of resistance to targeted therapies, including EGFR inhibitors, in NSCLC. Activation of alternative signaling pathways downstream of PI3K can occur, bypassing EGFR inhibition and leading to treatment failure. Targeting the PI3K pathway has thus emerged as a potential therapeutic strategy for NSCLC. Several inhibitors targeting different components of the PI3K pathway, such as PI3K, Akt, and mTOR, have been developed and evaluated in preclinical and clinical studies [33]. These inhibitors aim to disrupt the aberrant activation of the PI3K pathway, enhancing the efficacy of current NSCLC treatment approaches. In this study, KEGG analysis indicated a significant correlation between FDX1 overexpression and the PI3K pathway along with some regulators.

Furthermore, KRAS G12C mutations are prevalent in lung adenocarcinoma, comprising a significant proportion of cases. While recent advancements have led to the development of targeted therapies such as Sotorasib and Adagrasib, which specifically inhibit KRAS G12C, resistance to these treatments is emerging as a major issue. Several studies have implicated multiple pathways in mediating resistance to KRAS G12C inhibitors, highlighting the complex nature of drug resistance in this context. Given the central role of Ferredoxin 1 as a signaling mediator in lung adenocarcinoma tumorigenesis, it is plausible that it may also contribute to KRAS G12C resistance. Ferredoxin 1 has been implicated in various cellular processes, including metabolism, redox regulation, and signaling pathways, which are dysregulated in cancer cells. Therefore, investigating the involvement of Ferredoxin 1 in mediating resistance to KRAS G12C inhibitors could provide valuable insights into novel therapeutic strategies. By elucidating the potential interactions between Ferredoxin 1 and KRAS G12C signaling pathways, it can identify additional targets for combination therapies or alternative treatment approaches to overcome resistance and improve the efficacy of existing KRAS G12C inhibitors [34, 35]. Moreover, Metabolic reprogramming is a hallmark of cancer, enabling tumor cells to sustain rapid proliferation and survival under adverse conditions. KRAS mutant lung cancer, characterized by dysregulated KRAS signaling pathways, exhibits distinct metabolic alterations to meet the increased energy demands and biosynthetic requirements of proliferating cells. Ferredoxin 1, as a central mediator in cellular redox regulation and metabolism, likely plays a pivotal role in orchestrating these metabolic adaptations in KRAS mutant lung cancer. Previous studies have implicated Ferredoxin 1 in modulating various metabolic pathways, including glucose metabolism, lipid metabolism, and mitochondrial function, which are crucial for supporting tumor growth and survival. In the specific context of KRAS mutant lung cancer, aberrant KRAS signaling drives metabolic rewiring, promoting aerobic glycolysis (the Warburg effect), glutamine addiction, and altered lipid metabolism to fuel tumor progression. Ferredoxin 1 may act as a key regulator in these processes by modulating metabolic enzymes, redox-sensitive transcription factors, and signaling pathways involved in nutrient sensing and utilization [36, 37]. Understanding the interplay between Ferredoxin 1 and metabolic pathways in KRAS mutant lung cancer provides valuable insights into the underlying mechanisms driving tumorigenesis and identifies potential targets for therapeutic intervention. Targeting Ferredoxin 1-mediated metabolic vulnerabilities could offer novel strategies to selectively inhibit tumor growth while sparing normal cells, thereby improving the efficacy of cancer treatment and overcoming therapeutic resistance. Collectively, these aspects warrant further investigation and discussion in the context of our research on Ferredoxin 1 and its implications in lung adenocarcinoma progression.

## Conclusions

This study unveiled compelling evidence highlighting a substantial down-regulation of FDX1 in LUAD tissues. FDX1, emerging as a formidable tumor suppressor, wielded its influence by curbing the proliferation, migration, and invasion capacities of cancer cells. The findings tantalizingly hinted at a potential regulatory interplay between FDX1 and GPRIN2 in the intricate tapestry of LUAD progression. These results not only deepened but enriched our comprehension of the molecular mechanisms at play in LUAD, opening avenues for innovative therapeutic approaches targeting the dynamic duo of FDX1 and GPRIN2 in combatting this formidable variant of lung cancer. To unravel the full intricacies, further studies are imperative to illuminate the precise signaling pathways intricately woven into the FDX1-GPRIN2 axis, along with exploring its clinical relevance in the context of LUAD patients.

### Supplementary Information

Additional file 1: Figure S1. Validation of FDX1 stable overexpression A549 and H1975 cell lines using lentiviral vectors. **A** Validation of FDX1 mRNA overexpression efficiency in two NSCLC cell lines, A549 and H1975, through Real-time PCR. **B** Validation of FDX1 protein overexpression efficiency in the same cell lines through Western Blot and **C** immunofluorescence. ***P < 0.001 compared to OC-NC.

Additional file 2: Figure S2. GRRIN2 is down-regulated in LUAD tissues in GEPIA database. **A** GPRIN2 was down-regulated in LUAD tissues than in normal tissues, and **B** patients with lower GPRIN2 expression had a shorter survival, similar to FDX1. **C** In GEO database (GSE33532), it showed a significant down-regulation of GPRIN2 in LUAD tissues (n = 80) compared to adjacent normal tissues (n = 20). **D**, **E** The expression of FDX1 and GPRIN2 showed a positive correlation in the LUAD tissues in GEO database (GSE33532) and TCGA database. ^*^P < 0.05 and ^***^P < 0.001

## Data Availability

All data generated or analyzed during this study are included in this published article.
