# Supplementary figures and images for: Ferredoxin 1: a gatekeeper in halting lung adenocarcinoma progression through activation of the GPRIN2 signaling pathway
Source: J Transl Med. 2024 May 27;22:510. doi: 10.1186/s12967-024-05277-6 (PMC11131317; doi:10.1186/s12967-024-05277-6)

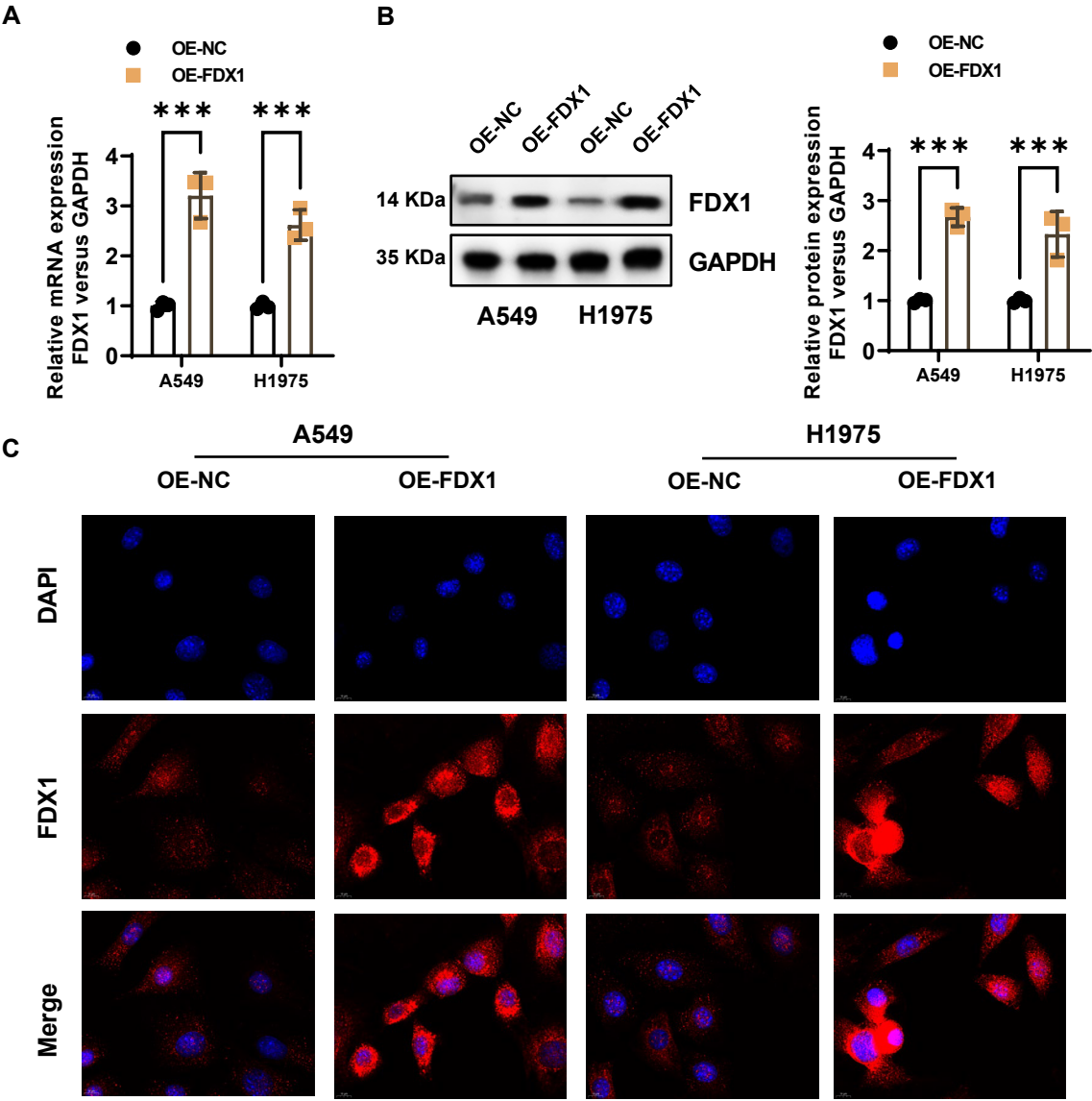

Supplement: Supplementary file 1 — Additional file 1: Figure S1. Validation of FDX1 stable overexpression A549 and H1975 cell lines using lentiviral vectors. A Validation of FDX1 mRNA overexpression efficiency in two NSCLC cell lines, A549 and H1975, through Real-time PCR. B Validation of FDX1 protein overexpression efficiency in the same cell lines through Western Blot and C immunofluorescence. ***P < 0.001 compared to OC-NC. [file 12967_2024_5277_MOESM1_ESM.pdf]

A

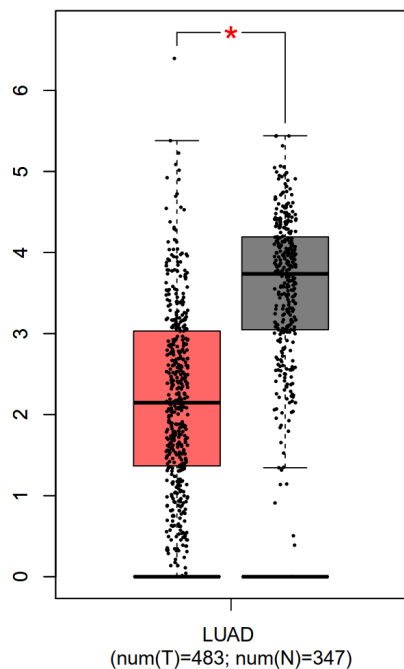

B

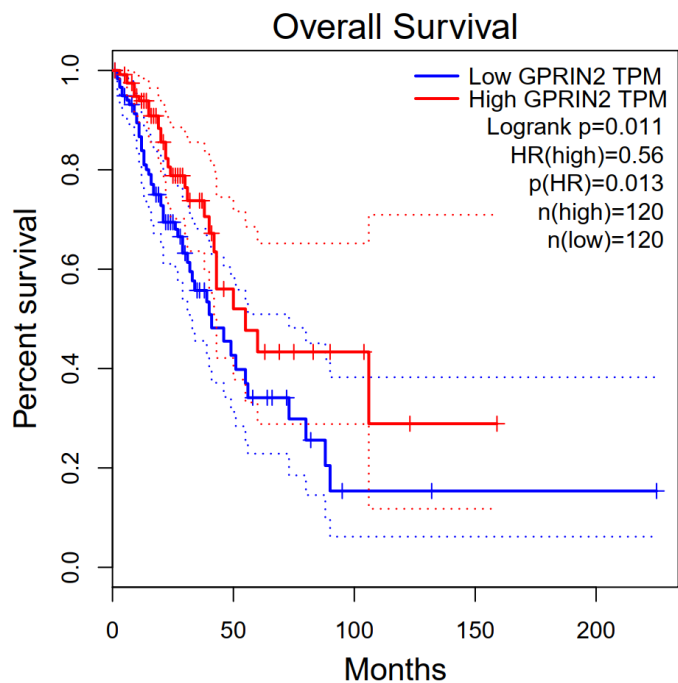

C

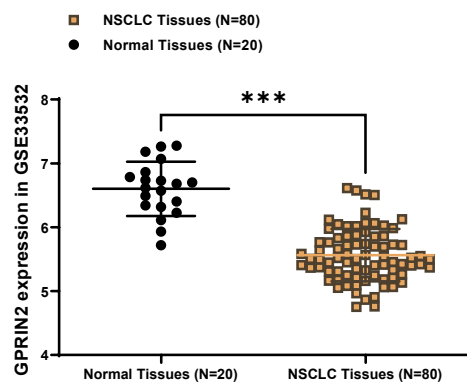

D

Correlation coefficient:  $1.60e-01$ ;  
 $P$ -value:  $1.56e-01$

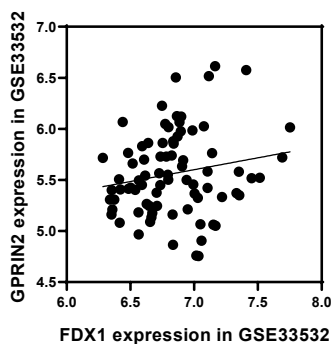

E

Correlation coefficient:  $4.55e-02$ ;  
 $P$ -value:  $3.03e-01$

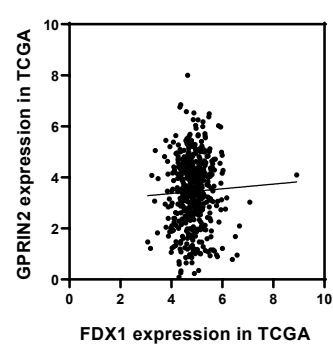

Supplement: Supplementary file 2 — Additional file 2: Figure S2. GRRIN2 is down-regulated in LUAD tissues in GEPIA database. A GPRIN2 was down-regulated in LUAD tissues than in normal tissues, and B patients with lower GPRIN2 expression had a shorter survival, similar to FDX1. C In GEO database (GSE33532), it showed a significant down-regulation of GPRIN2 in LUAD tissues (n = 80) compared to adjacent normal tissues (n = 20). D, E The expression of FDX1 and GPRIN2 showed a positive correlation in the LUAD tissues in GEO database (GSE33532) and TCGA database. *P < 0.05 and ***P < 0.001 [file 12967_2024_5277_MOESM2_ESM.pdf]
